# Characterization of the complete chloroplast genome of *Prunus clarofolia* C.K. Schneid (Rosaceae)

**DOI:** 10.1080/23802359.2021.1975513

**Published:** 2021-09-22

**Authors:** Yan-Feng Song, Qi Ye, Xian-Gui Yi, Xian-Rong Wang, Meng Li

**Affiliations:** Co-Innovation Center for Sustainable Forestry in Southern China, College of Biology and the Environment, Nanjing Forestry University, Jiangsu, Nanjing, China

**Keywords:** Chloroplast genome, *Prunus*, *Cerasus*

## Abstract

*Prunus clarofolia* is an endemic species that widely distributed in subtropical regions of China. Here we present its complete plastome. The plastome of *P. clarofolia* is successfully assembled from raw reads sequenced by Illumina Hiseq 2500 platform system. The complete chloroplast of this species is 158,053 bp in length with 36.7% overall GC content, including a pair of invert repeat regions (IR) (26,393bp) that is divided by a large single copy region (LSC) (86,088bp) and a small single copy region (SSC) (19,179bp). The plastid genome contained a total of 130 genes, including 85 coding genes, 8 rRNA genes, and 37 tRNA genes. Each of *rps*16, *atp*F, *rpo*C1, *clp*P, *pet*B, *pet*D, *rpl*16, *rpl*2, *ndh*B, and *ndh*A contains one intron, *rps*12 and *ycf*3 contains two introns. Phylogenetic analysis indicates that *P. clarofolia* has a closer relationship with *P. avium*.

*Prunus* L. subg. *Cerasus* (Mill.) A. Gray contains almost 150 species that mainly distribute in temperate and subtropical regions of the northern hemisphere (Yu et al. 1986). Recent studies have shown that frequent interspecific hybridizations and non-significant morphological differences have complicated the taxonomy of this subgenus (Ohta et al. 2007; Shi et al. 2013; Zhang et al. 2017). *Prunus clarofolia* C.K. Schneid (Fedde 1905) wildly spread in subtropical regions of China, and its leaves and bracts with sparse pubescent and umbellate inflorescences are often regarded as unique features. However, there still remains blank about the genetic relationship between this important wild species which are distributing in China and other subg. *Cerasus* species. Therefore, we sequenced the whole chloroplast genome of *P. clarofolia* to elucidate its phylogenetic relationship with other subg. *Cerasus* species.

The plant material was obtained from Songyang, Zhejiang province, China (28°87′56″N 119°28′35″E, altitude 357 m). A specimen was deposited at Nanjing Forestry University (https://shengwu.njfu.edu.cn/; collector: Meng Li, limeng@njfu.edu.cn; voucher number: NF: 161098736). Total DNA was extracted from fresh leaves with a modified CTAB protocol (Cai et al. 2014). The whole genome sequencing was conducted by Nanjing Genepioneer Biotechnologies Inc. (Nanjing, China) on the Illumina Hiseq 2500 platform (Illumina, San Diego, CA, USA). A total of 1.35 Gb of clean paired-end reads (Phred scores >20) were assembled using the programme GetOrganelle v1.7.5 (Jin et al. 2020). The plastome was annotated by the web application GeSeq (https://chlorobox.mpimp-golm.mpg.de/geseq.html) (Tillich et al. 2017).

The total length of complete chloroplast genome of *P. clarofolia* is 158,053 bp, with a total GC content of 36.7%. The plastid genome has a typical quadripartite structure, including large single-copy region (LSC) of 86,088bp, small single-copy region (SSC) of 19,179 bp, and a pair of inverted repeat regions (IRA and IRB) of 26,393bp each. The complete cp genome contains 130 genes, including 85 protein-coding genes, 37 tRNA, and. 8 rRNA genes. In this plastome genome, *rps*16, *atp*F, *rpo*C1, *clp*P, *pet*B, *pet*D, *rpl*16, *rpl*2, *ndh*B and *ndh*A contains an intron while *rps*12 and *ycf*3 contains 2 introns.

To determine the phylogenetic position of *P. clarofolia*, the complete chloroplast genome of *P. clarofolia* was aligned with other 28 subg. *Cerasus* species and 2 outgroups of subg. *Prunus* from GenBank using MAFFT v7.467 (Katoh et al. 2005) and visually checked and adjusted in Bioedit. Maximum-likelihood (ML) analysis was conducted in IQ-TREE v2.1.1 (Vergara et al. 2015) with 1000 bootstrap replications. The result was well-resolved and revealed that *P. clarofolia* was belonged to subg. *Cerasus* and was most closely related to *P. avium* (Figure 1). In summary, the complete plastid genome of *P. sargentii* will provide useful genetic information for increasing the richness of subg. *Cerasus*, as well as assisting in phylogenetic and evolutionary studies of subg. *Cerasus*.

**Figure 1.**
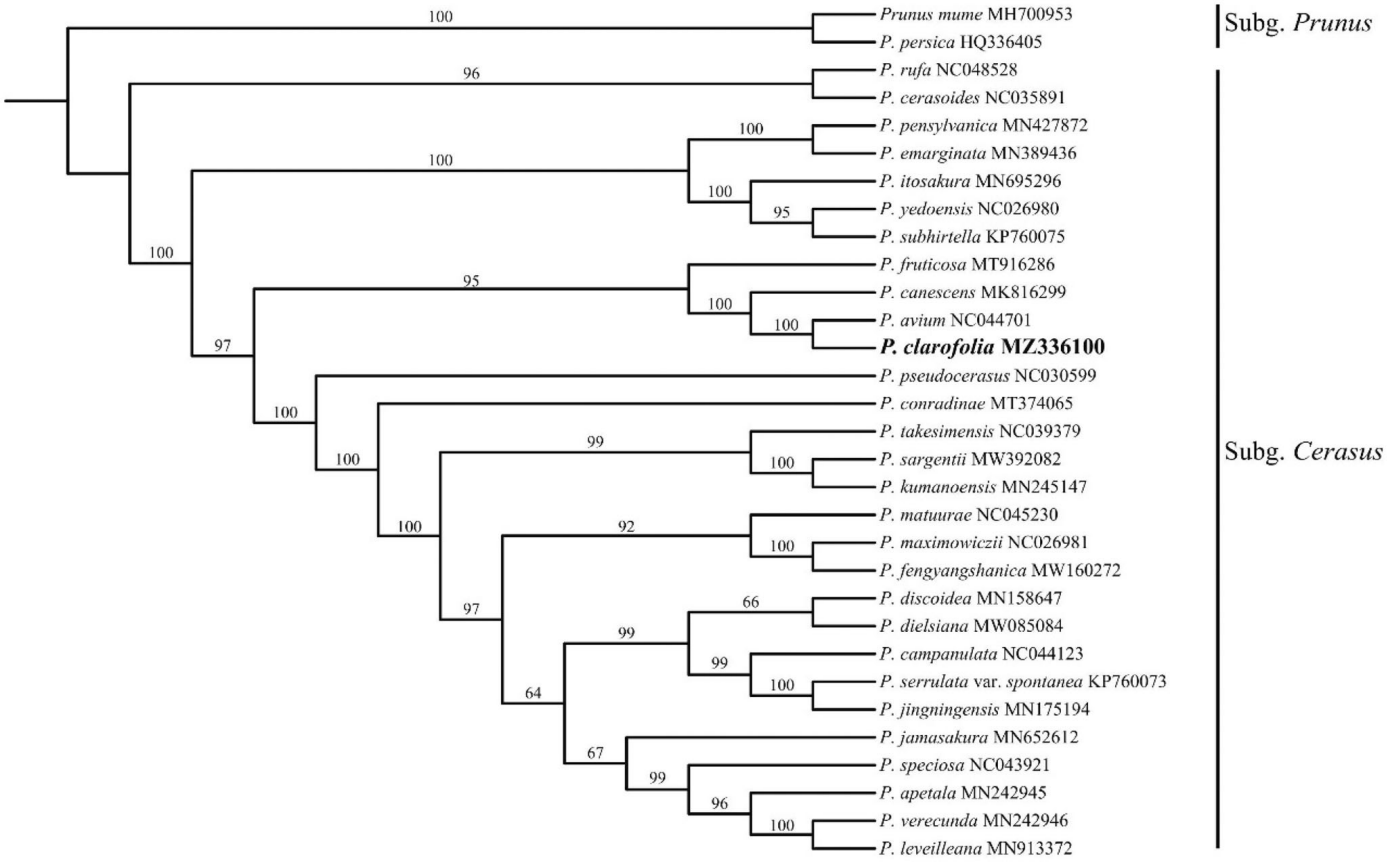
Maximum-likelihood phylogenetic tree for *P. clarofolia* based on 31 complete plastid genomes. *P. mume* and *P. persica* (Rosaceae) were used as outgroup and the support values are displayed above the branches.

## Data Availability

The plastid genome in this study is available in the NCBI GenBank (https://www.ncbi.nlm.nih.gov/genbank/) with an accession number MZ336100, and SRA submitted to NCBI under the BioProject No. PRJNA732592, Biosample NO. SAMN19324472, and SRA number: SRR14661355.
